# Effect of drying temperature of the dicalcium phosphate (DCP) on its X-ray diffraction patterns, spatial structure and solubility, retention of calcium and phosphorus, growth performance, tibia characteristics in broiler chickens fed diets supplemented with phytase

**DOI:** 10.1016/j.psj.2025.105831

**Published:** 2025-09-16

**Authors:** A. Mafi, S. Khalaji, M. Hedayati, F. Kaviani

**Affiliations:** a Department of Animal Science, Faculty of Agriculture, Malayer University, Malayer, Iran, 65719-95863; b Department of Clinical Sciences, Faculty of Veterinary Medicine, Bu-Ali Sina University, Hamedan, Iran

**Keywords:** Calcium, DCP, FESEM, Phosphorous, XRD

## Abstract

An experiment was conducted to evaluate the influence of the drying temperature of the di-calcium phosphate (**DCP**) on its X-ray diffraction patterns (**XRD**), spatial structure and solubility, retention of calcium and phosphorus, growth performance, tibia mineralization and strength in broiler chickens fed diets supplemented with phytase. A total of 500, 1-d-old Ross 308 female broiler chickens were randomly allocated to a 5  ×  2 factorial arrangement and fed diets contained DCPs dried at 5 different temperatures (60, 90, 110, 160 and 200°C) and two levels of phytase enzyme supplementation (0 or 1000 FYT/kg). There were 10 treatments and 5 cages with 10 chicks per each treatment. XRD pattern of DCP showed sharp peaks in lower temperature which indicting good crystallinity of DCP particles. Increasing the drying temperature resulted in amorphous solid with a XRD pattern typical for amorphous solid with reduced peaks height. Field emission scanning electron microscope (**FESEM**) images revealed considerable agglomeration and clustering of the DCP particles by increasing the drying temperature especially at 160 and 200°C compared to the DCP dried at 60°C. Solubility of DCP in 2 % citric acid (**CA**) was reduced by increasing the drying temperature linearly (*P* < 0.01). The solubility of DCPs dried at 60, 90, 110, 160 and 200°C were 96.2, 91.4, 88.1, 82.6 and 78.2 % respectively. Chicks fed diets supplemented with DCP dried at 160 and 200°C had lower **BW** and higher **FCR** (*P* ≤ 0.01) compared to the other chicks fed diet supplemented with DCP dried at 60, 90 and 110°C. Plasma Ca and P concentration was significantly higher (*P* ≤ 0.01) in chicks fed diets supplemented with DCP dried at 110°C on day 20. At day 30, plasma P concentration was significantly higher (*P* ≤ 0.01) in chicks fed diets supplemented with DCP dried at 60°C. Phytase supplementation had no significant effect on Ca, P and serum alkaline phosphatase (**ALP**) activity throughout the experiment. No significant difference in serum ALP activity were found among chicks fed different DCPs, however, intestinal ALP activity was higher (*P* ≤ 0.05) in chicks fed diets contain DCP dried at 110°C. Intestinal ALP activity was reduced significantly (*P* ≤ 0.05) by inclusion of exogenous phytase. Birds fed diet contained DCP dried at 60°C had the highest (P ≤ 0.01) level of P retention at both 10 and 32 days of age. Increasing the drying temperature of DCP reduced P retention significantly (P ≤ 0.05). Calcium retention were not affected (*P* > 0.05). The proximal length and proximal head thickness of femur bone, tibia ash and P content was higher (P ≤ 0.05) in birds fed diets contained DCP dried at 60°C. Tibia breaking strength was reduced in birds fed diets contained DCP dried at 160°C (P ≤ 0.05). In conclusion, the results of this study indeed showed that higher drying temperature of DCP negatively affect its structure and reduced its crystallinity and solubility which was illustrated by XRD diffraction patterns and FESM images.

## Introduction

Phosphate rock (Ca_10_(PO_4_)_6_F_2_) that can be classified as igneous apatite and sedimentary phosphorite according to their mineralization mechanism (Notholt et al., 1994). A geological survey reported that the world resources of phosphate rock are approximately 300 billion tons (United States Geological Survey, 2024). Phosphate rock resources occur principally as sedimentary marine phosphorites. It has been identified that large phosphate resources (almost 230 billion tons) are exist on the continental shelves and on seamounts in the Atlantic Ocean and the Pacific Ocean (United States Geological Survey, 2024). Consequently, world mine resources are almost 74 billion tons. Global production of phosphate rock and phosphate production capacity, in terms of P2O5 content, was estimated almost 220 and 69 million tons by 2027, respectively (Wu et al., 2018). It is obvious that phosphate rock reserves available to supply agricultural demands are diminishing severely in quality and quantity. Phosphate reserves that can be mined easily are estimated to about 12,000 million tons of rock according to reports by the FAO (2004). The currently operated mines have about 6,370 million tons of reserves and by 2030 these reserves will be diminished by half (Von Horn and Sartorius, 2009). Consequently, the phosphate price has jumped dramatically globally due to the capacity shortage and more difficult accessibility and the decreasing quality of the reserves which has been leads to higher processing costs (Von Horn and Sartorius, 2009).

Severe extraction of phosphate from phosphate rock mines can indeed lead to a decline in ore quality and an increase in impurities (Barshan et al., 2019). This is primarily due to the removal of higher-grade phosphate rock, leaving behind lower-grade material with a higher concentration of unwanted elements. Purity reduction of the phosphate rock by removing pure phosphate mineral can make the remaining rock more susceptible to water absorption or leaching due to changes in its chemical or physical properties. Furthermore, severe mining extraction can disturb the surrounding rock, potentially increasing its water content through increased surface area for water infiltration or by altering drainage pattern. Consequently, quality reduction and the presence of unwanted materials (impurities) and water in the phosphate rock delivered to the processing line can significantly reduce the value and the purity of the phosphoric acid and its P2O5 content to producing the DCP (Salas et al., 2017). Commonly, concentrated phosphoric acid, used commercially to produce DCP should contain around 54-62 % P2O5 (75-85 % H₃PO₄). The minimum acceptable P2O5 of phosphoric acid for DCP production is generally around 48 %. However, the P2O5 content of the phosphoric acid show considerable reduction and its water content and corrosivity has been increased in recent years due to the need for higher temperatures in the reactor accelerate and filtration stages during the phosphoric acid evaporation process because of impurities (Salas et al., 2017; Abdelouahhab et al., 2022).

Commonly, rotary drum dryer with a temperature of approximately 85 to 120°C has been used for DCP drying. Lower P2O5 (phosphorus pentoxide) and higher water content in phosphoric acid used for DCP production increases the need for addition of more acid and applying the higher temperature and longer drying time in dryer to produce a DCP with 17 % phosphate. Consequently, the increase in dryer temperature during the DCP production may affect the crystallinity, particle size, spatial structure and solubility. Therefore, in the following trial, DCP were processed using a commercial drier with different temperature to evaluate the effects of drying temperature on its crystallinity and molecular changes and its correlation to solubility and retention of Ca and P in broiler chicken.

## Materials and methods

### Ethics Statement

The experimental procedures were approved by the Ethical Animal Care and Use Committee of the University of Malayer (Malayer, Iran) and have been carried out based on procedures and guidelines (vol. 1. Isfahan University of Technology (Isfahan, Iran)) approved (ethical approval ID: IR.MUI.REC.1396.3.814) by the Animal Care Committee of the Iranian Council of Animal Care (1995).

### DCP production and drying

A phosphoric acid with 52.6 % P2O5 and limestone with 40 μm particle size were used to production of the test DCP. Prior to the DCP production, the P2O5 content of phosphoric acid and Ca content of limestone were determined using the methods described by Heydari et al. (2024). Briefly, total P2O5 content of phosphoric acid was quantified by adding the 0.4 ml Ammonium heptamolybdate (50 ml/liter) (Merck kGaA, 64271 Darmstadt, Germany), 0.4 ml Perchloric acid and 0.2 ml 1-Amino-2‑hydroxy-4-naphthalenesulfonic acid (CAS-No: 116-63-2, Merck kGaA, 64271 Darmstadt, Germany), and color intensity was read using a spectrophotometer (UNICO 2150, Germany) measuring absorbance at 700 nm (method 946.06, AOAC International, 2005), and the calcium were determined by titration using di-Ammonium oxalate monohydrate (50 ml/liter) (CAS-No: 6009-70-7, Merck kGaA, 64271 Darmstadt, Germany), and 0.02 M KMnO₄ after digestion of 1-g samples of limestone with 6 M HCl (5 ml) and 1 M HNO₃ (1 ml) at 250°C for 20 minutes (method 968.08 AOAC International, 2005). The particle size distribution and the geometric mean diameter (*μ*m) of limestone source were determined in triplicate according to the recommended procedure of ASABE (2007 and 2008). Briefly, 4 subsamples (100 g) were sieved through 9 sieves (sieve numbers 6, 8, 10, 14, 18, 40, 60, 120, 200) using a Filtra shaker (fritsch analysette 3 spartan, FRITSCH GmbH, Germany). For production of each batch of DCP with 17.5 % phosphorous and 24 percent calcium content, 1000 kg of phosphoric acid was mixed with 800 kg limestone using a twin shaft stainless steel paddle mixer (4000 l capacity) and then dried using a high efficiency DCP rotary drum dryer with 12 meter length and 1.5 meter diameter, fueled by gas using fired gas furnace with capacity of 350000 kcal/hour, passing capacity of 2 metric tons per hour and equipped with internal electronic thermometers at the beginning, middle and discharging area of the dryer, which was cleaned prior to each drying run. The flow rate of phosphoric acid and limestone into the mixer was controlled using the flow meter and feeder rate to have proper mixing and complete reaction. Dryer temperature was set to 60, 90, 110, 160 and 200°C at each specific run.

### Chemical Analyses, X-ray diffraction and Electron Microscopy

Produced DCPs were analyzed for P and Ca using the methods described by Heydari et al. (2024), crude ash (ISO 5984, 2002) and for DM (ISO 6496, 1998a). Briefly, total phosphorus content of the DCPs samples were determined by digestion of 1-g samples of DCP with 6 M HCl (5 ml) and 1 M HNO₃ (1 ml) at 250 °C for 20 minutes and phosphorus was quantified by adding the 0.4 ml Ammonium heptamolybdate (50 ml/liter) (Merck kGaA, 64271 Darmstadt, Germany), 0.4 ml Perchloric acid and 0.2 ml 1-Amino-2‑hydroxy-4-naphthalenesulfonic acid (CAS-No: 116-63-2, Merck kGaA, 64271 Darmstadt, Germany), and color intensity was read using a spectrophotometer (UNICO 2150, Germany) measuring absorbance at 700 nm (method 946.06, AOAC International, 2005), and the calcium content was measured by titration using di-Ammonium oxalate monohydrate (50 ml/liter) (CAS-No: 6009-70-7, Merck kGaA, 64271 Darmstadt, Germany), and 0.02 M KMnO₄ after digestion of 1-g samples of diets with 6 M HCl (5 ml) and 1 M HNO₃ (1 ml) at 250°C for 20 minutes (method 968.08 AOAC International, 2005).

A Unisantis XMD-300 X-ray powder diffractometer (Unisantis Europe GmbH, Germany) was used for XRD analysis (40 keV generator voltage; 30 mA tube emission current). When more complete XRD spectra were acquired, these were between 2*θ* of 10° to 88°, with a step size of 0.02° and a 1.5 s dwell time. Phase and peak identication were then performed using International Centre for Diffraction Data and Crystallography Open Database spectral libraries.

Surface morphology of DCP particles were analyzed using field emission scanning electron microscope (FESEM) (TESCAN MIRA4, TESCAN, Czech Republic). The DCP powder was mounted on the sample holder using double-sided adhesive carbon tape. The acceleration voltage was fixed to 10 kV. Scanning electron microscopy (SEM) images of test DSPs were made at a 3 magnification of 50,000 (1 µm), 100,000 (500 nm) and 250,000 (200 nm) times. The order of magnification was chosen such as to result in an optimal view of the spatial structure of all test products.

### Solubility in citric acid

Solubility of test DCPs were measured by modification and simplification of the method described by Sullivan et al. (1992). The method of Sullivan et al. (1992) for assessment and quantification of soluble phosphate of DCP has over the years proven useful to many research and industrial laboratories around the world. Unfortunately, it’s require multiple dilutions, filtrations and digestion unit makes the method time consuming to many modern laboratories. We have, therefore, modified Sullivan et al. (1992) original method by adapting it to present-day commercially rapid, simple, repeatable and user friendly method. This modified method was validated by comparing it to method proposed by Sullivan et al. (1992). Briefly, a sample (1 g) of test phosphates was weighed into a 250-mL volumetric flask. One hundred milliliters of 2 % CA was added to the sample. The samples were vigorously shaken for 30 min, brought to volume, mixed and 1-mL was transferred to a 100-mL volumetric flask, and the flask brought up to volume again. Then, 1-mL of sample was transferred to test tube and 4-mL water, 0.4 ml Ammonium heptamolybdate (50 ml/liter) (Merck kGaA, 64271 Darmstadt, Germany), 0.4 ml Perchloric acid and 0.2 ml 1-Amino-2‑hydroxy-4-naphthalenesulfonic acid (CAS-No: 116-63-2, Merck kGaA, 64271 Darmstadt, Germany), were added and were kept for around 15 min at room temperature in dark. Then the color intensity was read using a spectrophotometer (UNICO 2150, Germany) measuring absorbance at 700 nm. Phosphorus solubility calculated according to the total P of test DCPs.

### Bird husbandry and experimental diets

A total of 500, 1-d-old Ross 308 female broiler chickens with an initial BW of 45 ± 1.1 grams (parent stock age, 44 weeks) were obtained from a local hatchery and were housed in an environmentally controlled building from 1 to 42 days post-hatch. The chicks were divided into 50 battery cages with raised wire floors. The experiment was performed as a completely randomized design in a 5  ×  2 factorial arrangement with 5 different drying temperatures of DCP (60, 90, 110, 160 and 200°C) and two levels of phytase enzyme supplementation (0 or 1000 FYT/kg). There were 10 treatments and 5 cages with 10 chicks per each treatment. An experimental soy-maize mash diet with same levels of P and Ca from DCP dried at different temperature were formulated to be approximately isonitrogenous and isocaloric (ME based) for the starter (1-11 days post-hatch) and grower phases (12-24 days post-hatch) and finisher phases (25-42 days post-hatch) (Table 1). Feed and water were provided ad libitum during the experimental period. Body weight (BW) and cumulative feed intake (FI) were measured on 7, 21 and 42 days of age and feed conversion ratio (FCR) was calculated. FI in each cage for each period was calculated by total amount of feed placed in the feeders minus the residual feed. Chicks were brooded following standard temperature regimens, which gradually decreased from 32 to 24°C by day 20; the lighting cycle was 20-hour light and 4-hour dark.

**Table 1:** Composition and calculated analyzes of the basal diet.

Ingredients	One to 11 days	12 to 24 days	25 to 42 days
	Basal diet	Basal diet	Basal diet
Corn	518.2	560.5	598.8
Soybean meal, 420g/kg CP	410	360	325
Wheat bran	-	10	10
Soybean oil	27	32	35
Di-calcium phosphate	20	16	12
Limestone	9	8	6
Sodium chloride	3.5	2	2
Sodium bicarbonate	-	1	1.5
Vitamin and mineral premix^1^	5	5	5
DL – methionine, 990 g/kg	3.5	2.7	2.2
L- lysine HCl, 780 g/kg	2.3	1.8	1.5
L-threonine	0.5	-	-
Choline chloride	1	1	1
Calculated composition (g/kg)			
Metabolizable energy (MJ/kg)	12.45	12.64	12.97
Crude protein (CP)	22	20.5	19
Lys digestible	1.26	1.14	1.06
Met digestible	0.54	0.49	0.47
Met + Cys digestible	0.96	0.89	0.84
Thr digestible	0.84	0.76	0.71
Calcium	1	0.9	0.8
Total P	0.72	0.61	0.47
Available P	0.5	0.45	0.38
Sodium	0.18	0.16	0.16
Chlorine	0.19	0.17	0.17
(Na+*K*)-CL mEq/kg	243	227	205
Analyzed composition			
Crude protein (CP)	22.7	21.2	19.7
Calcium	1.07	0.93	0.87
Total P	0.71	0.64	0.58

1 Vitamin and mineral mix supplied the following per kg of diet: transretinol: 13 mg; cholecalciferol: 0.5 mg; a tocopherol acetate: 80 mg; menadione: 3 mg; thiamine: 3 mg, riboflavin: 8 mg; pyridoxine: 5 mg; cyanocobalamin: 0.024 mg; nicotinic acid: 60 mg; folic acid: 2 mg; Ca pantothenate: 15 mg; choline: 1000 mg; Mn: 120 mg; Zn: 1100 mg; Cu: 16 mg; Se: 0.3 mg; I: 1 mg; and Fe: 40 mg.^3^Sand was substituted (wt:wt) by fiber source.

### Blood characteristics

On d 20 and 30, two chicks were chosen and randomly picked from each pen and blood samples were taken 2 ml into heparin containing tubes by puncturing the brachial vein. The samples were centrifuged for 10 min at 3,000 × *g* at 20°C and the plasma were harvested and stored at −20°C for later analysis. Plasma calcium and phosphorus concentration were assayed using Sysmex KX-21 N automated hematology analyzer (KX-21 N, Sysmex Corp., Kobe, Japan) according to the method described by Robertson and Marshall (1979). Briefly, a plasma specimen, free of clot was collected into a bottle as quickly as possible to avoid hemolysis and elevation of serum phosphorus from hydrolysis or leakage of phosphate present in erythrocytes and then transferred into a bottle containing 10 mL of 10 percent v/v hydrochloric acid to avoid phosphate precipitation, adjusted to pH 2, diluted with distilled water and phosphorous was determined by direct method using Bionik Diagnostic kit (Lot no. 142374, Ref no. 3142327, Ireland (by reaction with ammonium molybdate as a phosphomolybdate and measuring the color intensity at wavelength 340 nm. Alkaline phosphatase activity was determined according to the procedure describe by Morgenstern et al. (1965) with an automatic biochemical analyzer (Hitachi 917, Boehringer Mannheim, Ingelheim am Rhein, Germany) using a Bionik Diagnostic kit (Lot no. 140289, Ref no. 3140226) and measuring color intensity at 405 nm.

### Digestive alkaline phosphatase activity

Digestive alkaline phosphatase activity were determined on day 25. Two chicks from each cage were chosen randomly, weighed, feed removed from feeders for 6 h to permit intestinal emptying of birds and then the chicks were slaughtered by cervical dislocation. The duodenum and a 10-cm segment of the jejunum adjacent to the distal pancreas, free of residual food, were removed and frozen in liquid nitrogen, until preparation for assay. The frozen intestine was partially thawed in a refrigerator at 4°C for 2 h. The samples were homogenized (dilution 1:5, w/v) in cold buffer (50 mM Tris–HCl, pH 8.0 containing 10 mM CaCl_2_) on ice at 11000 rpm for 2 min according to the procedure devised by Beyranvand et al. (2019). Thereafter, the homogenate was centrifuged at 14000 g for 45 min at 4°C. The supernatant was collected, and aliquots were stored at −80°C. Alkaline phosphatase activity was measured at 37°C by spectrophotometer (UNICO 2150, Germany) following the procedure described by Walter and Schutt (1974). Briefly, homogenates were incubated with p-nitrophenyl phosphate as substrate and the increase in absorbance was measured continuously over 30 min at 405 nm.

### Calcium and phosphorous retention

On d 7 and 29, feed was removed from feeders for 12 h to permit intestinal emptying of the birds. The experimental diets were offered to the birds *ad libitum* for 24 h and excreta were collected daily on day 10 through 12 and 32 through 34 by placing a tray beneath the chicks in cages. The collected excreta were pooled and then mixed before a subsample was taken. Spilled materials such as feathers were removed, and excreta were dried (105 °C) inside the oven (GmbH, d-91126, Germany). FI was calculated by the total amount of feed placed in feeder minus the residual feed. Excreta and feed samples were ground through a 1-mm screen. Samples were then used to determine dry matter content following drying at 105 °C for 24 h. Calcium and P contents of diets and excreta samples were determined using the methods described by Heydari et al. (2024) as explained in details earlier. The coefficients for P and Ca retention were calculated as the difference between the intake and the output of the respective nutrient and this was divided by the intake of the nutrient as proposed by Pirgozliev et al. (2019).

### Bone characteristics

Two chicks from each cage were chosen randomly on day 25, weighed and were slaughtered by cervical dislocation. Both femur and tibia bones were collected, deboned, and frozen at –20°C. After thawing and weighting, proximal length (cm), lateral cortex thickness (mm), and proximal head thickness (mm) at both the femoral and metatarsal side were measured using a digital caliper. For determination of tibia ash, calcium and phosphorus content, fat contamination was first removed from the tibia using n-hexane through a 36-h extraction procedure followed by a 36-h extraction with diethyl ether and subsequent drying of the bones at 100°C for 24 h. Tibia ash content was determined by combusting the bones in a muffle furnace for 4 h at 500°C. Phosphorus and calcium content of tibia were determined according to procedures described by Heydari et al. (2024) respectively, as mentioned earlier. Tibia bones were subjected to a three-point bending test (method described by Jungmann et al., 2007) using an All-Digital Electronic Universal Testing Machine (Santam Instrument Co. Model-MRT-5, serial no. 628415) according to the recommendations from the American Society of Agricultural Engineers shear and 3-point bending test of animal bone (standard S459). Maximum load to break the tibia (N), total energy to fracture, extension, elongation and stress were measured.

### Statistical methods

SAS Software version 9.1 was used for statistical analysis (SAS Institute Inc., Cary, NC, 2003) and data were analyzed at cage level (2003). Normality of the data were assessed based on model residuals and were tested using shapiro-wilk normality test by the UNIVARIATE procedure. Data were analyzed using the MIXED procedure with drying temperature and phytase as the main effects and including a 2-way interaction between the main effects using the following model:

Y=μ+DT+Phytase+DT×Phytase+ε

with *Y* = dependent variable, µ= overall mean, DT= fixed effect of drying temperature (60, 90, 110, 160 and 200°C) and Ԑ = residual error term.

There were an average of 10 birds in each pen as the experimental unit for performance characteristics and an average of 2 birds as the experimental unit for the other characteristics. BW was added to the model as a covariable for tibia characteristics. Distribution of the means and residuals were examined to verify model assumptions. Results are presented as lsmeans ± SEM. Differences between treatment means were tested using Tukey test. Statistical significance was declared at *P* ≤ 0.05.

## Results

### Chemical Analyses, X-ray diffraction and Electron Microscopy

The P content of DCPs dried at 60, 90, 110, 160 and 200°C were 17.6, 17.1, 17.7, 17.4 and 17.2 and Ca content were 22.2, 23.1, 22.8, 23.4 and 22.9 % respectively. Phosphorous and Ca content showed no significant differences among DCPs (*P* > 0.05).

**Fig. 1:**
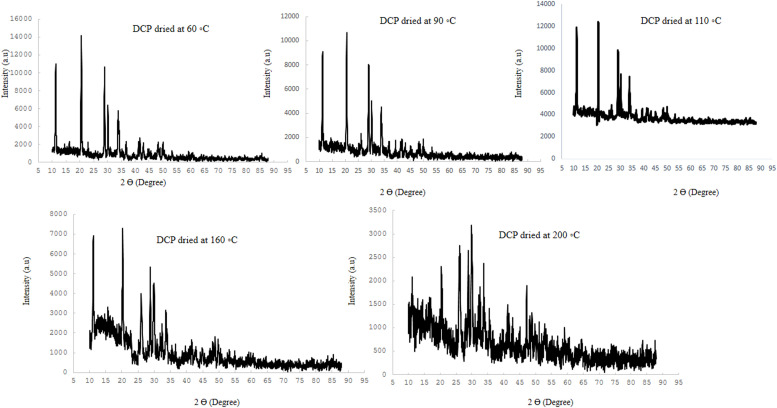
Typical XRD scan of DCP dried at different temperature; Detail of diffraction peaks in the 2*θ* range 10° to 88°.

**Fig. 2:**
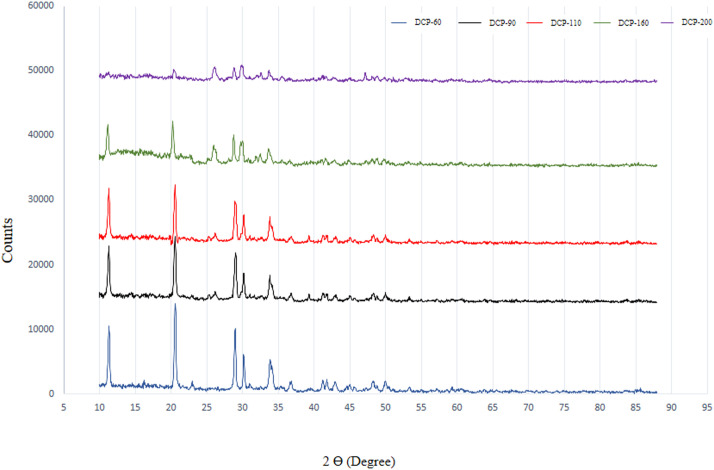
Variation with temperature of x-ray diffraction peaks for representative DCP, in the 10° to 88° region.

Typical XRD scan of DCP dried at different temperature and detail of diffraction peaks in the 2*θ* range 10° to 88° and also variation with temperature of x-ray diffraction peaks for representative DCP, in the 10° to 88° region are shown in Figs. 1 and 2. XRD pattern of DCP showed sharp peaks in lower temperature (60°C) such as that located at ∼11, 21 and 29° 2*θ* (with ∼intensity of 11000-14000 a.u) which indicting good crystallinity of DCP particles. Increasing the drying temperature resulted in amorphous solid with a XRD pattern typical for amorphous solid with linearly reduction of peaks such as that located at ∼12, 21 and 29° 2*θ* (with ∼intensity reduced blow the 3400 a.u). The variation of the XRD traces with temperature over the range from 60°C to 200°C for DCP samples are shown in Fig. 2. These were selected as they represent the extremes of changes of crystalline structure to amorphous samples by increasing the drying temperature. There is a systematic change in the peak height of some diffraction peaks such as peaks located at ∼11, 21 and 29° 2*θ* by increasing the drying temperature, respectively.

**Fig. 3:**
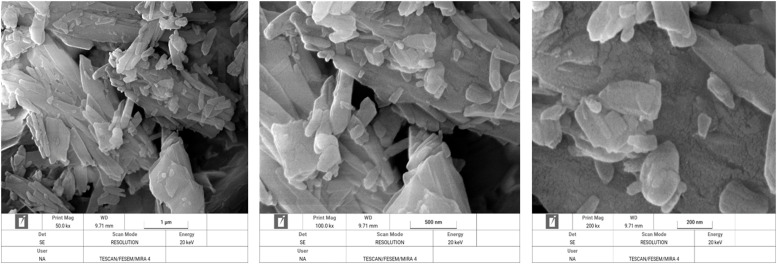
Scanning electron microscopy image of the DCP dried at 60°C a magnification of 50,000 (1 µm), 100,000 (500 nm) and 250,000 (200 nm) times.

In Figures 3 through 7 the FESEM images of the DCPs dried at different temperature are shown at a 3 magnification of 50,000 (1 µm), 100,000 (500 nm) and 250,000 (200 nm) for each specific dryer temperature. FESEM images revealed considerable agglomeration and clustering of the DCP particles by increasing the drying temperature especially at 160 and 200°C compared to the DCP dried at 60°C. FESEM also revealed high melting points of the particles at 200°C, which indicated a high degree of deformation at elevated temperatures.

### Solubility in citric acid

The solubility in citric acid for DCPs dried at different temperature is shown in Fig. 8. Results revealed linear (*P* ≤ 0.01) reduction in DCPs solubility by increasing the dryer temperature. The solubility of DCPs dried at 60, 90, 110, 160 and 200°C were 96.2, 91.4, 88.1, 82.6 and 78.2 % respectively.

### Growth performance

All growth performance parameters are presented in Table 2. At days 7 and 21, the chicks fed diets supplemented with DCP dried at 160 and 200°C had a lower BW and higher FCR (*P* ≤ 0.01) compared to the other chicks fed diet supplemented with DCP dried at 60, 90 and 110°C. Similarly, the FCR was higher (*P* ≤ 0.01) in chicks fed diets supplemented with DCP dried at 160 and 200°C compared to the other chicks fed diet supplemented with DCP dried at 60, 90 and 110°C at day 42. Average daily feed intake was not influenced by drying temperature of DCP throughout the experiment. Phytase supplementation had no significant effect on performance criteria at 7 days of age, however, BW was significantly higher (*P* ≤ 0.01) and FCR was lower (*P* ≤ 0.01) for birds received diets supplemented with phytase at days 21 and 42. Except for the FCR criteria at 42 days, no significant interactions between the drying temperature of DCP and phytase supplementation were observed in performance parameters throughout the experiment. On day 42, the lowest FCR was observed in birds fed DCP dried at 110 °C and supplemented with phytase and the higher FCR was observed in birds fed DCP dried at 160 °C and without phytase supplementation.

**Table 2:** Effects of drying temperature of DCP and phytase supplementation on performance characteristics at different ages.

Treatments		1-7 days			1-21 days			1-42 days		
		Ending Bird Weight (g)	FI^2^ (g)	FCR^3^	Ending Bird Weight (g)	FI^2^ (g)	FCR^3^	Ending Bird Weight (g)	FI^2^ (g)	FCR
Drying Temperature of DCP (°C)										
60		160^a^	154	0.964^b^	517^a^	711	1.377^c^	1927	3168	1.644
90		168^a^	157	0.937^b^	518^a^	725	1.400^c^	1929	3296	1.710
110		164^a^	162	0.990^b^	502^ab^	724	1.444^cb^	1946	3197	1.649
160		138^b^	160	1.161^a^	478^b^	733	1.532^a^	1883	3344	1.780
200		148^b^	163	1.096^a^	484^b^	716	1.480^ab^	1886	3287	1.744
	SEM	3.51	3.64	0.02	10.48	17.76	0.02	35.39	52.87	0.02
Phyatse (FYT/kg)										
0	154	160	1.045	488^b^	717	1.472^a^	1869^b^	3316	1.776	
1000	158	159	1.014	512^a^	727	1.421^b^	1959^a^	3276	1.675	
	SEM	3.22	2.30	0.02	6.63	11.24	0.02	22.38	33.44	0.02
Drying Temperature of DCP × Phytase										
60	0	157	156	0.995	504	704	1.400	1892	3173	1.677^bcde^
60	1000	163	152	0.934	530	718	1.354	1962	3157	1.609^cde^
90	0	164	156	0.948	504	717	1.422	1922	3297	1.716^abcd^
90	1000	171	159	0.927	531	732	1.377	1936	3295	1.705^bcd^
110	0	158	159	1.008	482	708	1.470	1874	3276	1.750^abc^
110	1000	170	165	0.972	523	741	1.418	2019	3118	1.547^e^
160	0	138	162	1.175	473	744	1.567	1783	3333	1.866^a^
160	1000	138	158	1.147	483	722	1.497	1983	3356	1.693^bcde^
200	0	150	165	1.099	475	713	1.502	1875	3322	1.773^abc^
200	1000	147	161	1.093	493	720	1.459	1897	3253	1.715^abcd^
	SEM	7.02	7.28	0.05	20.97	35.53	0.05	70.79	105.75	0.05
P-value										
Drying Temperature of DCP	0.001	0.468	0.001	0.033	0.923	0.007	0.642	0.257	0.010	
Phytase	0.190	0.787	0.200	0.014	0.558	0.020	0.007	0.399	0.001	
Drying Temperature of DCP × Phytase	0.619	0.799	0.959	0.870	0.868	0.994	0.295	0.741	0.042	

^a–d^Means within a column without a common superscript significantly differ (*P* < 0.05). ^1^FI = feed intake (g/period). ^2^FCR = feed conversion ratio (g of feed/g of body weigh gain).

### Blood characteristics

Effects of drying temperature of DCP and phytase supplementation on plasma Ca and P concentration and plasma ALP activity are presented in Table 3. Plasma Ca and P concentration was significantly higher (*P* ≤ 0.01) in chicks fed diets supplemented with DCP dried at 110°C at day 20. At day 30, plasma P concentration was significantly higher (*P* ≤ 0.01) in chicks fed diets supplemented with DCP dried at 60°C, whereas there was no significant difference in plasma Ca concentration among the chicks fed diets supplemented with DCP dried at different temperature. No significant difference in ALP activity were found at both days 20 and 30 among chicks fed diets supplemented with DCP dried at different temperature. Phytase supplementation had no significant effect on Ca, P and ALP activity throughout the experiment. There was a significant (P ≤ 0.05) interaction between the drying temperature of DCP and phytase supplementation on Ca concentration at 20 days of age. The highest plasma Ca concentration was observed in chicks fed diets contain DCP dried at 110 °C and supplemented with phytase and lowest plasma Ca was observed in chicks fed diets contain DCP dried at 60 °C without any phytase supplementation.

**Table 3:** Effects of drying temperature of DCP and phytase supplementation on plasma Ca and P content and plasma and digestive alkaline phosphatase activity at different age.

Treatments		20 d			30 d			25 d
		Plasma Ca (mg/dl)	Plasma P (mg/dl)	Plasma ALP (U/L)	Plasma Ca (mg/dl)	Plasma P (mg/dl)	Plasma ALP^1^ (U/L)	Intestinal ALP^1^ (U/L)
Drying Temperature of DCP (°C)								
60		5.66^b^	5.57^ab^	1125	8.16	7.09^a^	4130	6.3
90		6.18^b^	4.70^b^	1705	8.71	6.31^ab^	2928	6.6
110		8.17^a^	6.02^a^	1256	7.30	5.43^b^	2441	10.2
160		6.15^b^	5.74^ab^	1720	8.40	6.00^ab^	1770	6.8
200		6.42^b^	5.84^ab^	1727	7.75	5.40^b^	2638	6.1
	SEM	0.43	0.26	385	0.46	0.36	740	0.90
Phyatse (FYT/kg)								
0		6.39	5.72	1446	7.75	6.09	2610	8.2
1000		6.76	5.46	1578	8.38	6.00	2952	6.3
	SEM	0.27	0.23	243	0.29	0.23	468	0.50
Drying Temperature of DCP × Phytase								
60	0	5.35^d^	5.90	1157	8.15	7.45	4336	5.7^b^
60	1000	5.96^bcd^	5.24	1093	8.17	6.74	3924	7.0^b^
90	0	7.3^b^	5.70	1546	7.40	5.94	3772	7.7^b^
90	1000	5.36^d^	3.96	1824	10.02	6.67	2084	5.5^b^
110	0	7.25^bc^	5.68	968	6.90	5.26	1404	13.7^a^
110	1000	9.10^a^	6.37	1544	7.70	5.60	3477	6.7^b^
160	0	5.92^bcd^	5.92	1278	8.45	6.02	1873	7.2^b^
160	1000	6.37^bcd^	5.55	2309	8.35	5.97	1667	6.5^b^
200	0	6.10^bcd^	5.45	2236	7.85	5.77	1668	6.5^b^
200	1000	6.80^bcd^	6.36	1049	7.65	5.03	3608	5.7^b^
	SEM	0.87	0.73	770	0.93	0.74	1480	1.30
P-value								
Drying Temperature of DCP	0.002	0.012	0.689	0.253	0.016	0.268	0.024	
Phytase	0.317	0.555	0.674	0.140	0.802	0.609	0.031	
Drying Temperature of DCP × Phytase	0.050	0.108	0.340	0.196	0.561	0.330	0.046	

^a–d^Means within a column without a common superscript significantly differ (*P* < 0.05). 1ALP; alkaline phosphatase.

### Digestive alkaline phosphatase activity

Effects of drying temperature of DCP and phytase supplementation on digestive ALP activity are presented in Table 3. Intestinal ALP activity was higher (*P* ≤ 0.05) in chicks fed diets contained DCP dried at 110°C compared to the other treatment. Intestinal ALP activity was reduced significantly (*P* ≤ 0.05) by inclusion of exogenous phytase. There was a significant (P ≤ 0.05) interaction between the drying temperature of DCP and phytase supplementation on intestinal ALP activity at 25 days of age. The highest intestinal ALP activity was observed in chicks fed diet contained DCP dried at 110°C without any phytase supplementation.

### Calcium and phosphorous retention

Results of total tract retention of calcium and phosphorus at 10 and 32 days of age are presented in Table 4. Birds fed diet contain DCP dried at 60°C had the highest (P ≤ 0.01) level of P retention at both 10 and 32 days of age. Increasing the drying temperature of DCP reduced P retention significantly (P ≤ 0.05) at both 10 and 32 days of age. Calcium retention were not affected (*P* > 0.05) by drying temperature of the DCP throughout the experiment. Phytase supplementation had no effect on Ca and P retention during the trial. No significant interactions were observed between the drying temperature of DCP and phytase supplementation for Ca and P retention throughout the experiment.

**Table 4:** Effects of drying temperature of DCP and phytase supplementation on total tract retention of calcium and phosphorus at different age.

Treatments		10 d		32 d	
		Calcium	Phosphorous	Calcium	Phosphorous
Drying Temperature of DCP (°C)					
60		49.6	59.9^a^	47.4	45.9^a^
90		56.6	52.2^a^	62.8	34.2^b^
110		44.3	54.2^a^	58.5	32.6^b^
160		45.8	55.9^a^	51.7	39.0^b^
200		45.3	33.9^b^	53.9	37.5^b^
	SEM	4.97	2.70	5.55	4.12
Phyatse (FYT/kg)					
0	48.2	50.8	52.9	37.0	
1000	48.5	51.7	56.8	38.6	
	SEM	3.15	1.70	3.51	2.34
Drying Temperature of DCP × Phytase					
60	0	57.0	61.6	43.1	45.6
60	1000	42.2	58.3	51.7	46.4
90	0	63.7	54.1	60.7	30.9
90	1000	49.7	50.3	64.9	37.6
110	0	36.8	52.8	50.3	30.0
110	1000	51.8	55.6	66.6	35.2
160	0	41.9	53.0	50.0	41.9
160	1000	49.6	58.8	53.4	36.1
200	0	41.5	32.3	60.7	36.9
200	1000	49.1	35.5	47.2	38.1
	SEM	9.95	5.40	11.11	6.25
P-value					
Drying Temperature of DCP	0.404	0.001	0.349	0.001	
Phytase	0.944	0.703	0.451	0.413	
Drying Temperature of DCP × Phytase	0.137	0.646	0.438	0.286	

^a–d^Means within a column without a common superscript significantly differ (*P* < 0.05).

### Bone characteristics

Tables 5 and 6 presents the effects of drying temperature of DCP and phytase supplementation on femur, right and left tibia characteristics and on tibia breaking strength. As shown in Table 5, the proximal length and proximal head thickness of femur bone was higher (P ≤ 0.05) in birds fed diets contained DCP dried at 60°C. Drying temperature of DCP had no effect on fresh weight and lateral cortex thickness of femur bone, or on fresh weight, proximal length, proximal head thickness and lateral cortex thickness of left and right tibia bone (*P* > 0.05). Tibia ash and P content was reduced by increasing the drying temperature of DCP (P ≤ 0.05). Among the treatments, the highest tibia ash and P content was observed in birds fed diets contained DCP dried at 60°C. Phytase supplementation had no effect on femur, right and left tibia characteristics. A significant (P ≤ 0.05) interaction of DCP drying temperature with phytase was observed for lateral cortex thickness femur, right and left tibia. The lowest lateral cortex thickness femur, right and left tibia was observed in birds fed diets contained DCP dried at 160°C and supplement with phytase. No interaction between DCP drying temperature and phytase was observed for other measured characteristic of femur, right and left tibia.

**Table 5:** Effects of drying temperature of DCP and phytase supplementation on femur, right and left tibia characteristics.

Treatments		Femur				Right Tibia				Left Tibia						
		Fresh weight (g)	Proximal length (cm)	Proximal head thickness (mm)	Lateral cortex thickness (mm)	Fresh weight (g)	Proximal length (cm)	Proximal head thickness (mm)	Lateral cortex thickness (mm)	Fresh weight (g)	Proximal length (cm)	Proximal head thickness (mm)	Lateral cortex thickness (mm)	Ash (%)	Ca (%)	P (%)
Drying Temperature of DCP (°C)																
60		5.02	5.78^a^	10.01^a^	6.15	7.61	7.76	10.67	5.98	6.72	7.55	9.64	6.11	44.78^a^	37.37	19.02^a^
90		4.85	5.63^ab^	9.81^a^	6.28	7.46	7.63	10.42	5.91	7.30	7.54	9.55	5.95	41.70^ab^	38.00	16.90^ab^
110		4.76	5.57^b^	9.32^b^	6.23	7.33	7.58	10.33	5.92	7.04	7.57	9.52	5.94	41.81^ab^	37.12	16.57^b^
160		4.80	5.67^ab^	9.71^ab^	6.07	7.05	7.38	10.12	5.88	7.14	7.53	9.33	5.82	41.63^ab^	39.37	18.23^ab^
200		5.16	5.71^ab^	9.82^a^	6.28	7.53	7.65	10.07	5.80	7.42	7.65	9.61	5.80	40.86^b^	38.12	18.17^ab^
	SEM	0.21	0.05	0.14	0.13	0.28	0.21	0.23	0.12	0.31	0.07	0.25	0.13	1.07	0.87	0.73
Phyatse (FYT/kg)																
0		4.91	5.66	9.72	6.27	7.29	7.53	10.20	5.89	7.15	7.53	9.47	5.91	42.09	37.90	17.72
1000		4.93	5.69	9.74	6.14	7.50	7.67	10.44	5.91	7.10	7.61	9.58	5.94	42.23	38.10	17.83
	SEM	0.14	0.03	0.09	0.03	0.18	0.09	0.13	0.07	0.07	0.05	0.16	0.08	0.68	0.55	0.47
Drying Temperature of DCP × Phytase																
60	0	4.95	5.77	10.10	6.25^bcd^	7.40	7.72	10.42	5.72^cde^	6.42	7.48	9.38	5.83^bcde^	44.43	36.00	19.32
60	1000	5.09	5.80	9.93	6.05^bcd^	7.82	7.80	10.92	6.25^ab^	7.01	7.62	9.90	6.38^a^	45.14	38.75	18.73
90	0	5.05	5.65	10.08	6.60^a^	7.67	7.62	10.57	6.00^abcd^	7.86	7.60	9.53	6.07^abc^	42.04	38.50	17.79
90	1000	4.66	5.62	9.54	5.97^bcd^	7.25	7.65	10.27	5.82^abcde^	6.75	7.48	9.57	5.83^bcde^	41.35	37.50	16.02
110	0	4.65	5.52	9.08	6.05^bcd^	6.76	7.52	9.75	5.75^bcde^	6.85	7.47	9.15	5.83^bcde^	41.52	38.12	16.46
110	1000	4.87	5.62	9.56	6.42^b^	7.89	7.65	10.49	6.10^abc^	7.23	7.67	9.88	6.05^abcd^	42.11	36.12	16.67
160	0	5.06	5.72	9.81	6.27^bcd^	7.31	7.22	10.16	6.32^a^	7.58	7.60	9.94	6.14^ab^	42.40	40.12	18.31
160	1000	4.54	5.62	9.62	5.87^d^	6.80	7.55	9.98	5.45^e^	6.70	7.47	8.72	5.49^e^	40.85	38.62	18.14
200	0	4.85	5.62	9.56	6.17^bcd^	7.31	7.57	10.11	5.65^cde^	7.05	7.50	9.38	5.67^bcde^	40.03	36.75	16.73
200	1000	5.47	5.80	10.08	6.40^bc^	7.75	7.72	10.56	5.95^abcde^	7.79	7.80	9.84	5.93^abcde^	41.68	39.50	19.61
	SEM	0.43	0.11	0.29	0.25	0.56	0.29	0.41	0.25	0.62	0.15	0.51	0.25	2.15	1.75	1.47
P-value																
Drying Temperature of DCP	0.662	0.048	0.032	0.707	0.668	0.492	0.872	0.262	0.562	0.804	0.923	0.445	0.012	0.442	0.013	
Phytase	0.921	0.475	0.883	0.275	0.405	0.295	0.833	0.200	0.835	0.232	0.640	0.813	0.885	0.800	0.865	
Drying Temperature of DCP × Phytase	0.334	0.434	0.070	0.045	0.230	0.961	0.326	0.002	0.127	0.165	0.073	0.019	0.848	0.152	0.274	

^a–d^Means within a column without a common superscript significantly differ (*P* < 0.05).

**Table 6:** Effects of drying temperature of DCP and phytase supplementation on tibia breaking strength.

Treatments		32 d				
		Tibia breaking strength (N)	Tibia energy to fracture (J)	Extension (mm)	Elongation (%)	Stress (MPa)
Drying Temperature of DCP (°C)						
60		102^a^	116	4.51	4.59	2.42
90		101^a^	192	4.61	6.15	2.41
110		105^a^	136	4.76	6.35	2.50
160		64^b^	162	4.22	5.63	2.22
200		100^a^	158	4.12	5.50	2.37
	SEM	11.81	34.98	0.29	0.76	0.11
Phyatse (FYT/kg)						
0		89	147	4.54	5.49	2.40
1000		99	158	4.35	5.80	2.36
	SEM	8.10	22.12	0.18	0.48	0.07
Drying Temperature of DCP × Phytase						
60	0	95	143	4.46	3.10	2.26^ab^
60	1000	109	90	4.56	6.09	2.59^ab^
90	0	110	220	4.85	6.47	2.63^ab^
90	1000	92	163	4.37	5.83	2.19^ab^
110	0	113	84	5.15	6.87	2.70^a^
110	1000	96	187	4.37	5.83	2.30^ab^
160	0	43	168	4.37	5.83	2.41^ab^
160	1000	86	155	4.08	5.44	2.04^b^
200	0	86	121	3.88	5.18	2.04^b^
200	1000	113	195	4.37	5.83	2.70^a^
	SEM	25.63	69.96	0.58	1.52	0.23
P-value						
Drying Temperature of DCP	0.016	0.621	0.520	0.531	0.566	
Phytase	0.398	0.728	0.460	0.651	0.678	
Drying Temperature of DCP × Phytase	0.366	0.367	0.583	0.361	0.004	

^a–d^Means within a column without a common superscript significantly differ (*P* < 0.05).

As shown in Table 6, among the treatments, the tibia breaking strength was lower in birds fed diets contain DCP dried at 160°C (P ≤ 0.05). Other tibia criteria showed no differences regarding to the DCP drying temperature. Phytase supplementation had no effect on tibia breaking strength variables. Except for stress, there were no DCP drying temperature and phytase interactions (*P* > 0.05) for any of the variables of tibia breaking strength.

## Discussion

Phosphate rock reserves shortage for production of high quality phosphoric acid is a global challenge for industries (FAO, 2004). Besides industrial disturbances of low quality phosphoric acid with low levels of P2O5 and high water content which leading to poor quality of di-calcium phosphate products (Salas et al., 2017; Abdelouahhab et al., 2022), such products are associated with decreased DCP solubility and P digestion in bird’s intestine which would lead to increase in bone disorders and abnormalities and increase in phosphorous waste into the environments. In this study, the total Ca and P content of produced DCPs which was analyzed by digestion of DCP in strong HCl and nitric acids using the approved methods (Sullivan et al., 1992) was similar among the DCPs dried at different temperatures. The similar content of the total Ca and P of DCPs dried at different temperature which was acquires from the common analysis indicate that the drying temperature did not affect the total content of Ca and P and do not reflects the quality of the DCP for poultry.

**Fig. 4:**
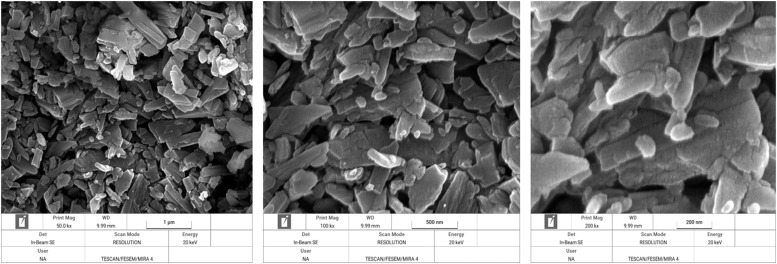
Scanning electron microscopy image of the DCP dried at 90°C a magnification of 50,000 (1 µm), 100,000 (500 nm) and 250,000 (200 nm) times.

**Fig. 5:**
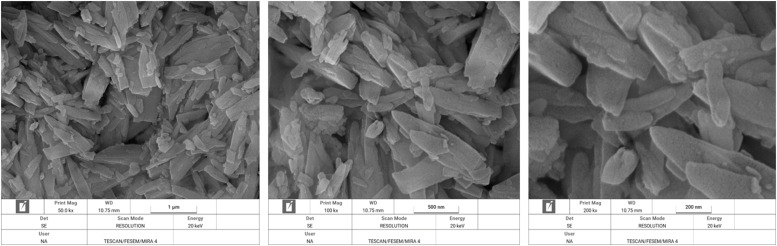
Scanning electron microscopy image of the DCP dried at 110°C a magnification of 50,000 (1 µm), 100,000 (500 nm) and 250,000 (200 nm) times.

**Fig. 6:**
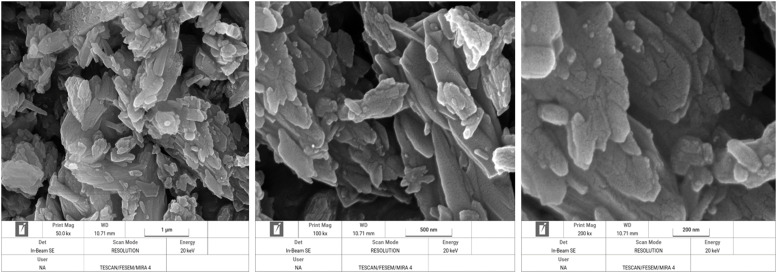
Scanning electron microscopy image of the DCP dried at 160°C a magnification of 50,000 (1 µm), 100,000 (500 nm) and 250,000 (200 nm) times.

**Fig. 7:**
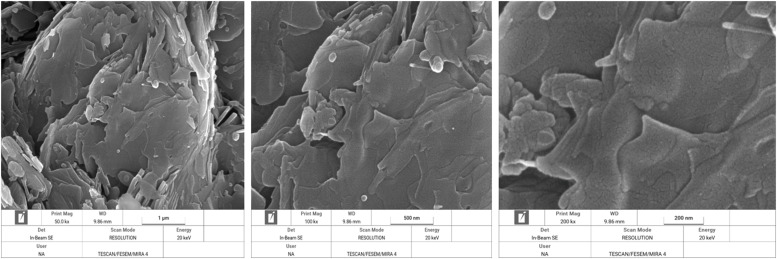
Scanning electron microscopy image of the DCP dried at 200°C a magnification of 50,000 (1 µm), 100,000 (500 nm) and 250,000 (200 nm) times.

XRD pattern results from the present study revealed that produced DCPs in this trial showed sharp peaks in lower temperature (60°C) such as that located at ∼11, 21 and 29° 2*θ* (with ∼intensity of 11000-14000 a.u) which they represent the extremes of high amount of crystal plane of DCP particles. The results indicate that increasing the drying temperature resulted in amorphous solid with a XRD pattern typical for amorphous solid with linearly reduction of peaks such as that located at ∼12, 21 and 29° 2*θ* (with ∼intensity reduced blow the 3400 a.u), which suggesting XRD pattern as a sensitive indicator for evaluating the DCP structural changes from high soluble crystalline particle to low soluble amorphous particles due to the drying temperature levels. To the knowledge of the authors, no other studies have been published that have studied the effect of drying temperature of DCP on its XRD pattern and digestibility of P. Furthermore, based on a visual interpretation of the FESEM images of the DCPs dried at different temperature, it seems that the spatial structure of the DCPs as shown by the FESEM images (Figures 3, 4, 5, 6, 7) can be related to the observed DCP solubility and its P retention. It seems that, based on visual inspection of the FESEM images, a high amount of low-size crystalline particles exist in DCPs dried at lower temperature (60°C) compared to the large, agglomerated and melted particles in DCPs dried at higher temperature (specially 160 and 200°C), which suggests that a compound with a large quantity of very small crystals with the higher surface area and the higher degree of crystallinity could increase the DCP solubility and its P retention as observed in current trial. An explanation for this relation is that the larger surface area in low size and crystalline particles may enhance solubilization of P in the small intestinal tract and subsequently improve intestinal absorption of P (Van Harn et al., 2017). The present study revealed that the solubility DCPs dried at lower temperature (60°C; 96.2 %) was almost 18 % higher than the solubility of DCPs dried at higher temperature (200°C; 78.2 %).

**Fig. 8:**
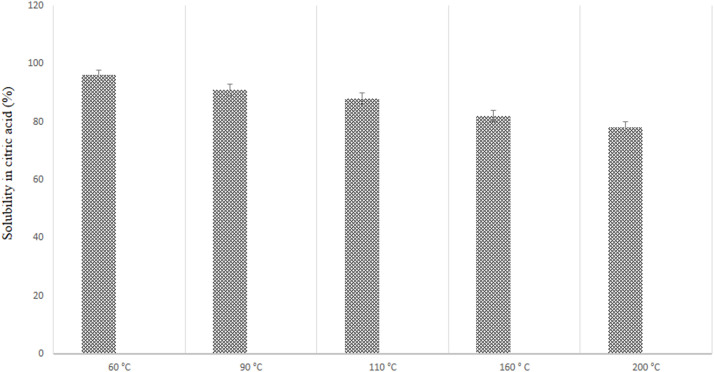
The solubility of DCP dried at different temperature in a 2 % citric acid.

Results from the present study showed that the increase in drying temperature of DCP negatively affected the body weight gain and FCR at 7 and 21 days of age. Many studies demonstrated that either a Ca and P deficiency or a reduction in their availability and utilization can impair broiler growth and bone development parameters particularly during early growth stages (Williams et al., 2000a; Rama Rao et al., 2003; Liu et al., 2017; Li et al., 2020). Insufficient intake, digestion and absorption of Ca or P, when the deficiency of one of them interferes with homeostasis of second one, results in retarded growth rate and poor bone mineralization (Shafey et al., 1990; Hurwitz et al., 1995).

Li et al. (2020) reported that the Ca and P levels in serum can reflect the nutritional status, digestion, absorption and utilization of Ca and P sources and bone resorption in broilers. In our present study, broilers fed diets contain DCP dried at 110°C had higher Ca and P concentration at day 20. However, at day 30, plasma P concentration was higher in chicks fed diets supplemented with DCP dried at 60°C and increasing the drying temperature of DCP reduced plasma P concentration. Plasma Ca levels was similar among the treatments. The depressed serum P level may be a result of reduction of the DCP solubility and P available for absorption in the gut for DCPs dried at higher temperature. However, it is well suggested (Zhang et al., 2020) that there was an important point that changes of serum Ca and P levels were in concert with Ca and P contents of diets, tibias and femurs. No differences in plasma Ca levels by aging the birds in chicks fed diets with DCPs dried at higher temperature might be explained with the compensatory role of bone resorption. Proszkowiec-Weglarz and Angel (2013) suggested that, low serum P concentration can leads to the activation of osteoclasts that, in turn, leads to increased bone resorption for maintaining both serum Ca and P level. Subsequently, a severe bone resorption can depresses the tibia ash and mineral content for maintaining serum Ca homeostasis, which could partly explain the similar serum Ca level. In addition, the results from the current study demonstrated that tibia ash and P content of broilers concomitantly showed strong responses to drying temperature, which both was reduced by increasing the DCP drying temperature. No significant difference in ALP activity were found at both days 20 and 30 among chicks fed diets supplemented with DCP dried at different temperature. It is well demonstrated that serum alkaline phosphatase (ALP) increase typically in responds to low Ca and P levels, as ALP is involved in bone turnover and the body attempts to restore normal calcium and phosphate levels (Zhang et al., 2020; Mehri et al., 2025). No response in serum ALP level in current trial might be due to the adequate levels of Ca and P in diets, which enabled broilers to maintain serum Ca and inorganic P levels within the bird’s requirement. The results of the present study showed that the Ca (5-9 mg/dl) and P (4-7 mg/dl) concentration of chicks among the treatments in current trail were within a range of normal serum Ca (6-11 mg/dl) and P (4-7 mg/dl) levels in broiler chickens.

In the present study, we found that intestinal ALP activity was higher in chicks fed diets contained DCP dried at 110°C compared to the other treatment. Intestinal ALP activity was reduced by inclusion of exogenous phytase. In addition, significant interaction effect was observed for intestinal ALP activity, in that the highest intestinal ALP activity was observed in chicks fed diet contained DCP dried at higher temperature without any phytase supplementation. The high levels of supplemented Ca and P and inclusion of phyatse can increase Ca and P concentrations in intestinal lumen and can led to higher pre-cecal P digestibility in chicks. Consequently, this can led to higher Ca and P concentrations in the ileum of and reduce the need for higher activation of intestinal ALP activity (Walk et al., 2018; Novotny et al., 2023). The higher intestinal ALP activity caused by the supplementation of DCP dried at 90, 110, 160 and 200°C without supplementation of exogenous phytase might be due to the lower concentration of Ca and P in intestinal lumen resulted from lower solubility of DCP and high concentrations of IP6 in intestine, which Walk et al. (2018) hypothesized is due to a triggering mechanism of IP6 degradation. Similar to the treatments without phytase supplementation, the contribution of mucosal phosphatase to phytate degradation is unclear (Novotny et al., 2023).

Based on the previous reports (Van der Klis and Versteegh, 1992; Shastak et al., 2012; Van Harn et al., 2017), there was an astonishing variation in the digestibility of phosphourous in anhydrate DCP. Van Harn et al. (2017) reported a 82.4 % pre-cecal phosphorus digestibility for anhydrate DCP which was substantially higher than results obtained by Van der Klis and Versteegh (1992) and Van der Klis and Versteegh (1993) who measured total tract P digestibility values of anhydrate DCP of 53.0 and 56.9 %, respectively. Additionally, lower anhydrate DCP digestibility levels were reported by Shastak et al. (2012) of 25 to 30 %. This huge variation might partially be due to the levels of drying temperature of DCPs used in these trials. The previous trials did not take into account the drying temperature which is a critical oversight for DCP solubility. This highlights the critical importance of Ca and P utilization efficiently by broilers when the DCP of the fed diets dried at lower temperature. A significantly reduction of P retention by increasing the drying temperature of DCP in current trial at both days 10 (59.9 vs 33.9; low vs high temperature) and 32 (45.9 vs 37.5; low vs high temperature) evidently represent the adverse effect of higher temperature on P digestibility, and based on the above findings, which was associated with structural changes of high soluble crystalline particles to less soluble amorphous particles that illustrated with XRD pattern and FESM images.

Femur proximal length, tibia ash and P content was higher in chicks fed diet contained DCP dried at 60°C. Additionally, the lowest lateral cortex thickness femur, right and left tibia and tibia breaking strength was observed in birds fed diets contained DCP dried at 160°C and supplement with phytase. Guinotte et al. (1991) reported that organic sourced Ca and P in broiler chicken diets resulted in higher tibia proximal length and lateral cortex thickness. Scott et al. (1982) reported that lower availability of minerals led to shorter tibia proximal length, shorter lateral cortex thickness, and malformations on tibia. Ca and P are primarily essential for bone mineralization (Rath et al., 1999; Blake and Fogelman, 2002), and increasing Ca and P in broiler chicken diets or their availability might positively influence bone mineralization and bone mineral content, leading to increase in bone strength (Driver et al., 2005a,b; Létourneau-Montminy et al., 2008). Previous studies have reported that calcium and phosphorus deficiencies negatively impact tibial performance in broilers (Jiang et al., 2016; Liu et al., 2016; Williams et al., 2000a; Li et al., 2025). It is well demonstrated that tibial breaking strength in broilers is closely related to dietary Ca and P levels, with higher levels increasing compressive strength (Christensen et al., 2003; Imari et al., 2020; Li et al., 2025). However, the present study showed reduced tibia breaking strength in chicks fed diets contained DCP dried at 160°C and not 200°C. This result might be due to the adequate levels of DCP in diets and inclusion of the phytase without considering its matrix value for P, which enabled broilers to maintain serum inorganic P levels within the bird’s requirement. This suggests that the reduction of DCP levels in broiler diet by applying the low temperature for drying the DCP could help to reduce the P levels in diets and reduces its wastage to environment. Güz et al. (2019) proposed that instead of increasing dietary inorganic Ca and P content, changing the source of Ca and P or increasing the bio-availability of the mineral phosphate sources might work in the same way.

## Conclusion

In conclusion, the results of this study indeed showed that higher drying temperature of DCP negatively affect its structure and reduced the its crystallinity and solubility which was illustrated by XRD diffraction patterns and FESM images. Higher drying temperature of DCP impaired growth and phosphorous retention and negatively impact tibial mineral content and its strength in some aspects. However, due to the adequate levels of P in diets, inclusion of the phytase showed no considerable interaction for much of the criteria measured in this trial, but in the case that the higher drying temperature of DCP which affects its quality may enhance the phytase usage with this kind of DCPs in diet and its inclusion may influence the Ca and P metabolism and bone characteristic when lower Ca and P levels were applying in practical diets, but further studies will be needed to elucidate the mechanism of action.

## CRediT authorship contribution statement

**A. Mafi:** Project administration, Investigation, Data curation. **S. Khalaji:** Writing – review & editing, Writing – original draft, Supervision, Software, Project administration, Investigation, Data curation, Conceptualization. **M. Hedayati:** Supervision, Methodology. **F. Kaviani:** Investigation, Formal analysis.

## Disclosures

For the manuscript entitled “Effect of drying temperature of the dicalcium phosphate (DCP) on its X-ray diffraction patterns, spatial structure and solubility, retention of calcium and phosphorus, growth performance, tibia mineralization and strength in broiler chickens fed diets supplemented with phytase”

A. Mafi, S. Khalaji, M. Hedayati, F. Kaviani

There is No conflict of interest
